# Mechanical Performance of Carbon-Fiber Geogrid-Reinforced Asphalt Pavement Systems Under High-, Low-Temperature, and Shear Loadings

**DOI:** 10.3390/polym18101161

**Published:** 2026-05-08

**Authors:** Jian Liu, Qi Wang, Zhiqiang Wang, Guangqing Yang

**Affiliations:** 1 Cangzhou Qugang Expressway Construction Co., Ltd., Cangzhou 061000, China; wzcf231914@163.com (J.L.); gtsyang@163.com (Q.W.); 2 School of Traffic and Transportation, Shijiazhuang Tiedao University, Shijiazhuang 050043, China; 3 Key Laboratory of Roads and Railway Engineering Safety Control of Ministry of Education, Shijiazhuang Tiedao University, Shijiazhuang 050043, China; yanggq@stdu.edu.cn; 4 Hebei Engineering Research Center on Application of Geosynthetics, Shijiazhuang Tiedao University, Shijiazhuang 050043, China; 5 School of Civil Engineering, Shijiazhuang Tiedao University, Shijiazhuang 050043, China

**Keywords:** road engineering, surface combined body, carbon fiber geogrid, rutting resistance, low-temperature cracking resistance, interlayer shear performance

## Abstract

The application of carbon-fiber-based geogrids in asphalt pavements is still in the nascent phase of research in China. Compared with glass fiber, carbon fiber undergoes processes such as electrochemical surface oxidation and coating with a sizing agent (polyurethane-based) to enhance its bond strength with bitumen or concrete, and to improve its wear resistance and suitability for construction. Utilizing a suite of laboratory tests including rutting tests, low-temperature flexural failure tests, and Leutner shear tests, this study researches the impacts of surface combined body type and geogrid type on the high- and low-temperature performance characteristics and interlayer shear performance of asphalt pavement structures. The results demonstrate that carbon-fiber-based geogrid reinforcement improves the rutting and low-temperature cracking resistance of asphalt surface combined bodies, with the carbon fiber geogrid (CCF) variant exhibiting superior performance to the carbon/glass fiber composite geogrid (GCF) in both aspects. Relative to GCF reinforcement, CCF reinforcement achieves increases of 12.80–13.74%, 4.53%, and 37.47% in dynamic stability, flexural tensile strength, and flexural tensile strength enhancement rate, respectively, indicating that the polymer coating process enhances the reinforcement effect of carbon-fiber-based geogrids. Carbon-fiber-based geogrid reinforcement compromises the interlayer shear performance of asphalt pavement composites; nevertheless, CCF reinforcement delivers 13.94–28.14% better interlayer shear performance than GCF reinforcement. This indicates that the polymer coating process enhances the shear resistance at the interface of carbon-fiber-based geogrids. Surface combined body type is a key factor governing the high- and low-temperature performance and interlayer shear behavior of reinforced surface combined bodies. The dynamic stability, maximum flexural-tensile strain, and interlayer shear strength of the AC-20/AC-25 are all superior to those of the AC-13/AC-20, with respective increases of 40.25%, 27.58%, and 8.5–25.6%. The test results may provide meaningful insights into the performance behavior of geogrid-reinforced asphalt pavements.

## 1. Introduction

Road pavements can be classified into three primary categories: flexible pavements, rigid pavements, and composite pavements [1,2]. Flexible pavements are pavements with low stiffness and low tensile strength that primarily rely on compressive and shear strength to withstand vehicle loads. In engineering practice, flexible pavements mainly include asphalt pavements and unbound crushed stone pavements. Owing to their distinctive material properties, asphalt pavements exhibit a distinct advantage in the construction of high-grade highways and have thus been widely adopted in engineering practice. However, the primary distress modes currently afflicting asphalt pavements include rutting, cracking, and fatigue damage [3,4,5,6,7]. To address the aforementioned challenges, relevant research efforts have primarily focused on optimizing aggregate gradation, deploying high-performance asphalt mixtures (e.g., modified asphalt binders, fiber additives, etc.), increasing the thickness of pavement surface layers, and incorporating reinforcing materials (such as geosynthetics) at the interface between semi-rigid bases and asphalt surface layers [2,8,9,10], with such approaches yielding certain positive outcomes. However, relevant studies have indicated that both increasing the thickness of the surface course and utilizing high-performance asphalt mixtures incur substantial economic costs, thus requiring case-specific selection in engineering practice. The incorporation of geosynthetic materials at the interface between the base course and surface course, or between adjacent asphalt surface courses, constitutes a cost-effective technical solution. Geogrids, in particular, exhibit excellent structural stability and remarkable environmental adaptability in field applications. Therefore, the interposition of geosynthetic materials at the interfaces between asphalt pavement layers represents an effective technical approach to mitigating distress-related issues in asphalt pavements [11,12].

Extensive research has been performed to examine the rutting resistance of geosynthetic-reinforced asphalt pavements. When deployed as reinforcing materials within granular base courses, geosynthetics have been proven effective in mitigating rutting distress in asphalt pavements and enhancing the overall structural performance of asphalt pavements [13,14,15]. Lee et al. [6], Wang et al. [8], Khodaii et al. [15], and Lee et al. [16] have collectively demonstrated the efficacy of geogrid reinforcement in improving the rutting resistance of asphalt pavements. Lee et al. [16] examined the effects of geosynthetic type and temperature on the rutting resistance of asphalt mixtures, concluding that different reinforcement configurations lead to distinct shear flow behavior. Lee et al. [6] evaluated the rutting resistance of fiber-reinforced asphalt overlay pavements through finite element numerical simulations, as well as laboratory and field tests. Their findings indicated that glass fiber and carbon fiber geogrids demonstrate comparable performance in mitigating rutting. Additionally, some studies have indicated that geogrid reinforcement contributes to a reduction in the permanent deformation of asphalt concrete [3,17]. Currently, rutting tests are widely adopted as the primary means of assessing the rutting resistance of asphalt mixtures worldwide [1,18]. The aforementioned studies mainly explore the rutting resistance of geosynthetic-reinforced asphalt overlays. However, research on the rutting resistance of new carbon-fiber-based geogrid-reinforced asphalt surface combined bodies remains scarce.

With respect to the low-temperature cracking resistance of geosynthetic-reinforced asphalt pavements, the primary influencing factors identified in the literature include geosynthetic type, reinforcement position, temperature, load category, existing surface course type, crack width, and asphalt mixture type [2,15,19,20,21]. Wang et al. [22] introduced a novel method for assessing the cracking resistance of reinforced asphalt mixtures, utilizing temperature-induced reflective cracking tests under flexural-tensile mode. Fereidoon et al. [19] and Irene et al. [20] evaluated the effects of geosynthetic type and modulus on the cracking resistance of reinforced asphalt mixtures. Saride et al. [23] explored the cracking resistance of geosynthetic-reinforced asphalt overlays by means of digital image correlation (DIC) and four-point bending fatigue tests. Lee et al. [6] examined the long-term performance of geogrid-reinforced asphalt overlays through finite element numerical simulations, as well as laboratory and field tests, and demonstrated that placing geogrids beneath the overlay effectively suppresses interfacial crack propagation. Behnia et al. [24] investigated the low-temperature cracking resistance of fiber-reinforced asphalt concrete through compacted disk tensile tests, indirect tensile tests, and acoustic emission tests, and found that the addition of fibers enhanced the low-temperature cracking performance of asphalt mixtures. In addition, several studies have shown that geogrid reinforcement has no marked influence on the crack initiation phase of reinforced asphalt mixtures, whereas it exerts a significant effect in the propagation phase by retarding crack expansion [25,26]. Taken together, the above studies indicate that research has mainly focused on the cracking resistance of geosynthetic-reinforced asphalt pavements, particularly those reinforced with glass-fiber geogrids. However, research on the cracking resistance of carbon-fiber geogrid-reinforced asphalt pavements remains limited.

The use of interlayer geosynthetic reinforcement [2,4] has been found to render the reinforced asphalt layers structurally weak, thereby reducing the service life of the pavement [27,28]. Currently, research on the interlayer shear behavior of geosynthetic-reinforced asphalt mixtures remains limited. Interlayer shear performance is governed by geosynthetic type (material, stiffness, aperture size), asphalt mixture characteristics, interlayer condition (bonding agent, temperature), shear loading rate, and aggregate morphology [1,25,28,29,30]. Existing studies indicate that using geosynthetics as interlayer reinforcement can reduce interlayer bond strength and shear strength [1,28,29,31]. Nithin et al. [27] investigated the effect of geosynthetic stiffness on interlayer shear strength of asphalt pavement using the Leutner shear test and found that shear-strength gains increased with decreasing geosynthetic tensile modulus. Wang et al. [2] evaluated the effects of test method, surface combined body type, and geogrid type on interlayer shear strength using shear tests. The results identified the test method as the most statistically significant factor influencing interlayer shear strength, followed by geogrid type. Walubita et al. [29] and Pasetto et al. [11] investigated the effect of geogrid type on the interlayer shear strength of asphalt pavements by means of shear tests. Lorenzo et al. [32] evaluated the effect of geogrid type on interlayer shear strength using a modified large-scale direct-shear apparatus and found that increasing geogrid tensile modulus does not necessarily enhance interlayer shear strength. Correia et al. [28] investigated the effects of geogrid characteristics on interlayer shear strength of asphalt pavement through combined field investigations and laboratory tests. The results indicated that geogrid properties influence shear strength more than tensile strength. The above studies mainly examine how the geosynthetic type affects interlayer shear behavior, with limited attention to carbon fiber geogrid-reinforced asphalt pavements.

Asphalt components, aging extent, fiber type, and temperature are also factors affecting the mechanical properties of asphalt mixtures, including rutting resistance and low-temperature cracking resistance [33,34,35,36,37]. Büchner et al. [38] investigated the mixing behavior of three different rejuvenators under various rheological parameters measured using a dynamic shear rheometer (DSR), proposed the required rejuvenator dosages needed to achieve target bonding performance at high and medium temperatures under various aging conditions, and evaluated their suitability for use in asphalt pavement rejuvenation applications. Ren et al. [39] studied the effects of asphalt aging and rejuvenator type on the creep behavior of asphalt mixtures using creep tests and found that long-term aging increases the shear stress, creep time, and residual stress ratio of the asphalt. Sun et al. [40] investigated the impacts of climate-induced temperature rise on aging characteristics, long-term service performance, and maintenance costs of asphalt pavements. They revealed that for each 1 °C rise in average temperature, the asphalt pavement aging rate accelerated by roughly 1.5–2.6%, accompanied by a 1.9–3.8% growth in total maintenance costs. Among all investigated cities, the polymer-modified 70R0 mixture presented the minimum overall maintenance expenditure. It is widely acknowledged that fiber incorporation into asphalt binders can optimize the viscoelastic properties of asphalt concrete mixtures and pavement structures, enhance moisture resistance, creep performance, and abrasion resistance, and suppress crack propagation. Meanwhile, fibers also improve low-temperature cracking resistance, flexural modulus, toughness, and tensile strength of mixtures, and notably elevate the fracture toughness of hot-mix asphalt (HMA). Lin et al. [41] investigated the influences of lignin fiber and polyester fiber on cracking resistance, fatigue performance, and rutting stability of high-content SBS modified asphalt (HCPMA) mixtures under unaged and aged states. The results showed that both fibers achieved enhanced reinforcement efficiency after short-term and long-term aging. Given its favorable interfacial compatibility with asphalt binders and superior mechanical performance, carbon fiber has been widely regarded as a promising modifier for asphalt pavement materials. Alfalah et al. [42] investigated the influences of fiber type on volumetric parameters and laboratory performance of asphalt mixtures, and revealed that carbon fibers can markedly enhance the anti-cracking performance of asphalt mixtures. Pirmohammad et al. [43] studied the effects of carbon fiber and kenaf fiber on the fracture properties of asphalt concrete. The results demonstrated that carbon fiber-reinforced asphalt concrete possessed better fracture performance than kenaf fiber-reinforced asphalt concrete. Jin et al. [44] investigated the performance of warm-mix asphalt (WMA) modified with waste tire rubber and nylon fiber composite (R-F) by adopting the mechanistic-empirical (M-E) pavement design method combined with laboratory tests. The results revealed that the R-F composite can effectively reduce the international roughness index (IRI) and enhance the cracking and rutting resistance of WMA. Jin et al. [45] studied the durability and aging resistance of hot-mix asphalt (HMA) containing tire fabric fibers and ground tire rubber. Their results demonstrated that adding tire fabric fibers can effectively improve the rutting and cracking resistance of HMA.

Despite the outstanding reinforcement benefits of carbon fiber, its utilization cost is markedly greater than that of natural fibers [46]. A major factor contributing to the high manufacturing cost of carbon fiber is the expensive precursor yarn, which occupies over 50% of its overall production expenditure [46,47]. Adopting low-cost textile-grade polyacrylonitrile (PAN) fiber precursors (Tex PAN) represents a feasible approach to cutting down the production cost of carbon fibers [9,46,48]. In recent years, numerous scholars have fabricated low-cost carbon fibers using Tex PAN precursors via specialized pre- and post-stabilization treatments, which markedly lowers the overall manufacturing cost of carbon fibers [46,48,49].

Based on the above analysis, this study aims to comprehensively evaluate the high- and low-temperature performance and interlayer shear behavior of asphalt pavement structures reinforced with carbon-fiber-based geogrids. This study investigated the effects of geogrid type (carbon-fiber geogrid and glass/carbon-fiber composite geogrid) and dense-graded asphalt concrete mixture (AC) surface combined body type (AC-13/AC-20 and AC-20/AC-25) on the rutting resistance, low-temperature cracking resistance, and interlayer shear behavior of asphalt pavement surface combined bodies. The findings of this study contribute to improved understanding of the performance characteristics of carbon-fiber-based geogrid reinforced asphalt pavement structures, thereby supporting the development of optimized design methodologies.

## 2. Materials and Methods

### 2.1. Materials

The two types of geogrids used in this study were a glass/carbon-fiber composite geogrid (denoted as GCF) and a carbon-fiber geogrid (denoted as CCF). The GCF has glass-fiber longitudinal ribs and carbon-fiber transverse ribs. The geogrids were supplied by Shandong Road New Materials Co., Ltd. (Tai’an, China). Key technical specifications are shown in Table 1. Compared with glass fiber, carbon fiber undergoes processes such as electrochemical surface oxidation and coating with a sizing agent (polyurethane-based) to enhance its bond strength with bitumen or concrete, and to improve its wear resistance and suitability for construction.

**Figure 1:**
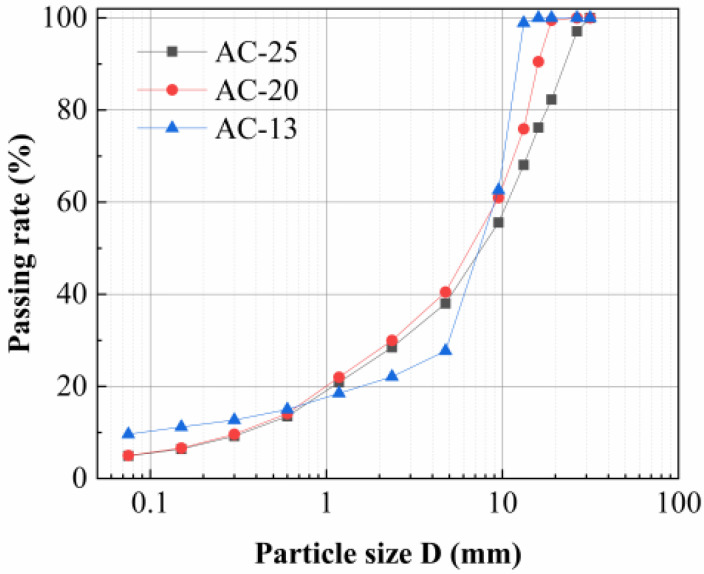
Asphalt mixture gradation curve diagram.

The asphalt utilized in this study was styrene–butadiene–styrene block copolymer (SBS)-modified asphalt. In accordance with Chinese Standard JTG E20, AASHTO T324-14, or CEN EN 12697-22:2020 [50,51,52], the technical parameters of SBS-modified bitumen were obtained through testing, as listed in Table 2. The tack coat adopted was PCR cationic emulsified asphalt, applied at a dosage of 0.4 L/m^2^ to achieve effective bonding between the upper and lower asphalt mixture layers, as shown in Table 3. The asphalt mixtures employed in this study comprised AC-13 (upper layer), AC-20 (middle layer), and AC-25 (lower layer), with the respective aggregate gradation curves illustrated in Figure 1. The AC-13 utilized a combination of basalt and limestone aggregates. According to practical engineering conditions of asphalt pavements in China [53,54,55], basalt was selected as coarse aggregates with nominal particle sizes of 10–15 mm and 5–10 mm, while limestone was used for the remaining aggregate fractions. The AC-20 and AC-25 were formulated exclusively with limestone aggregates. The mineral powder was limestone powder. The asphalt aggregate ratios for AC-13, AC-20, and AC-25 mixtures were determined using the Marshall method, as summarized in Table 4.

### 2.2. Surface Combined Body

In this study, the surface combined body refers to a two-layered asphalt pavement structure, comprising upper and lower asphalt mixture layers bonded by a tack-coat interface. Asphalt pavement structures are typically divided into upper, middle, and lower layers. According to composite theory and field interface configurations, surface combined bodies are defined as upper-layer–middle-layer and middle-layer–lower-layer systems. The adopted surface combined body type not only better represents actual asphalt pavement structures but is also more suitable for construction practice. The surface combined body types adopted in this study encompass two variants, namely the AC-13/AC-20 and AC-20/AC-25.

### 2.3. Specimen Preparation

**Figure 2:**
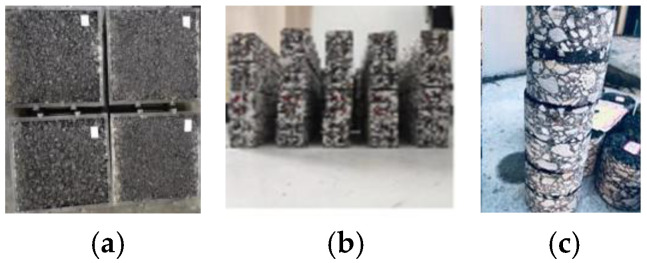
The partial trabecular specimens: (**a**) rutting plate; (**b**) beam specimen; (**c**) cylindrical specimen.

The preparation steps for the rutting specimen of the reinforced surface combined body were as follows. (1) Preparation of lower-layer asphalt mixture: the test mold dimensions used were 300 mm long × 300 mm wide × 50 mm thick. The lower-layer asphalt mixture was prepared in accordance with the requirements of the Chinese standard JTG E-20. (2) Layout of carbon-fiber-based geogrid: After demolding, the lower molded specimen was transferred to a large test mold (300 mm × 300 mm × 100 mm). The surface was cleaned, SBS-modified emulsified asphalt was uniformly applied, and the geogrid was laid. (3) Preparation of upper-layer asphalt mixture: the upper-layer asphalt mixture was prepared using the same method as the lower layer. Upon cooling to room temperature, the composite rutting plate was obtained. The specimen shown in Figure 2a was subjected to rutting tests.

The preparation protocol for composite beam specimens of the reinforced surface combined body was described as follows. The rutting plates were cut into beam specimens with prescribed dimensions of 250 mm × 47 mm × 50 mm, and all specimens were prepared accordingly. The specimen illustrated in Figure 2b was subjected to low-temperature bending failure tests.

The preparation procedure for cylindrical specimens of the reinforced surface combined body was as follows. After the prepared rutting plates were cured for 72 h, three cylindrical specimens, each 100 mm in diameter and height, were extracted from each plate by core drilling. Specimens were subjected to shear testing as illustrated in Figure 2c.

### 2.4. Test Type

(1) Rutting test

**Figure 3:**
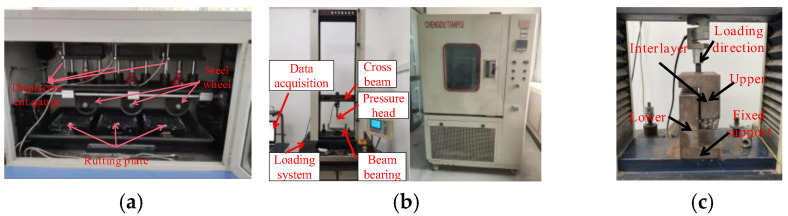
The test equipment: (**a**) rutting test; (**b**) low-temperature bending failure test [9]; (**c**) Leutner shear test.

The rutting test was carried out on the LHCZ-9 three-lane fully automatic rutting tester, as illustrated in Figure 3a. The device was provided by Beijing Lanhang Zhongke Measurement and Control Technology Research Institute (Beijing, China).The wheel load of 0.7 MPa at a loading frequency of 42 ± 1 times/min was applied at a constant temperature of 60 ± 1 °C. The test was terminated either upon reaching a testing duration of 1 h or when the maximum deformation of the specimen reached 25 mm.

(2) Low-temperature bending failure test

The low-temperature bending failure test was carried out using a WDW-1020 microprocessor-controlled electronic universal testing machine and a WD-402 high-low temperature test chamber. The devices were provided by the Institute of Testing Instruments at Changchun Kexin Company, Chinese Academy of Sciences (Changchun, China).The beam specimens were tested at −10 °C under displacement control at a rate of 50 mm·min^−1^. The test was terminated when the load dropped to 80% of the peak load. The experimental setup is shown in Figure 3b.

(3) Leutner shear test

The Leutner shear test was performed by applying a constant shear rate parallel to the interlaminar interface, with the experimental setup illustrated in Figure 3c. In this study, the shear displacement rate was set at a constant 2.54 mm/min. All tests were performed at a controlled temperature of 20 °C on specimens with a diameter of 100 mm. Prior to testing, all specimens were subjected to isothermal treatment at the designated test temperature for 12 h. The test was terminated when shear stress dropped to 60% of the peak shear stress [2].

## 3. Results and Discussion

### 3.1. Rutting Resistance

#### 3.1.1. Influence of Geogrid Type and Surface Combined Body Type on the Maximum Rut Depth

**Figure 4:**
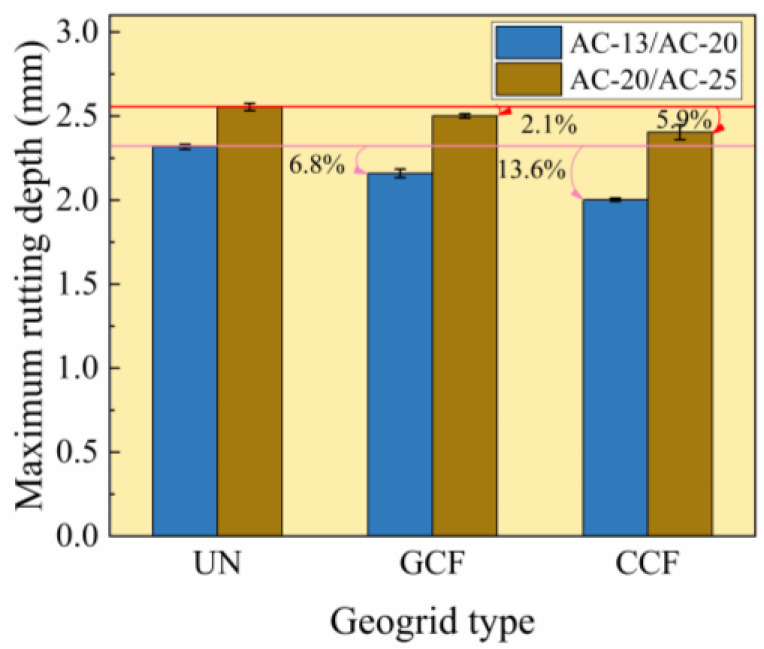
Maximum rutting depth test results.

Figure 4 shows the test results of the maximum rutting depth of various geogrid-reinforced surface combined bodies. As shown in Figure 4, under the AC-20/AC-25 conditions, the maximum rutting depth of the reinforced surface combined body is reduced by 2.1–5.9% compared with the unreinforced specimen. Under the AC-13/AC-20 conditions, the maximum rutting depth of the reinforced surface combined body is reduced by 6.8–13.6% compared with the unreinforced specimen. This confirms that the geogrid-reinforced composite exhibits a higher load-bearing capacity under identical conditions. The underlying reason is that rutting is primarily controlled by the shear strength of the asphalt mixture [56]. Geogrid reinforcement enhances confinement of the asphalt surface combined body, thereby improving its shear strength. The maximum rutting depth of GCF is consistently greater than that of CCF, at approximately 1.04–1.08 times the value for CCF. This observation is attributed to the performance difference between CCF and GCF, primarily due to differences in longitudinal rib properties. Longitudinal ribs are critical for improving transverse rib flexural stiffness and maintaining geogrid integrity [8,57]. Improved longitudinal rib performance results in greater reinforcing efficiency of the geogrid and superior rutting resistance of the surface combined body.

As can also be seen from Figure 4, under identical reinforcement conditions, the reduction in maximum rutting depth of AC-20/AC-25 is less than that of AC-13/AC-20. Under the condition of CCF reinforcement, the reduction in maximum rutting depth increases by 30% when the surface combined body changes from AC-20/AC-25 to AC-13/AC-20. Under the condition of GCF reinforcement, the reduction in maximum rutting depth increases by 100% when the surface combined body changes from AC-20/AC-25 to AC-13/AC-20. This indicates that the surface combined body type significantly reduces the maximum rutting depth of the reinforced surface combined body. Based on the above analysis, CCF exhibits better overall performance when combined with the surface combined body. However, considering economic efficiency, GCF is suggested as the preferred reinforcement material for the surface combined body.

#### 3.1.2. Influence of Geogrid Type and Surface Combined Body Type on Dynamic Stability

**Figure 5:**
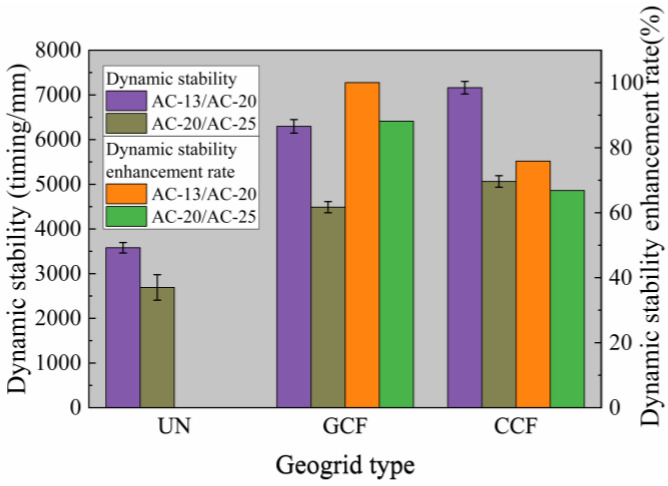
Dynamic stability and its enhancement ratio test results.

Dynamic stability is adopted as the core evaluation index for rutting tests, and its calculation formula is presented in Equation (1) [58]. Figure 5 shows the test results for the dynamic stability of various geogrid-reinforced surface combined bodies. To further compare the influence of the two geogrids (CCF and GCF) on the dynamic stability of reinforced surface-layer composites and quantify their reinforcement efficiency, the enhancement ratio of dynamic stability (*ER_DS_*) [9] is introduced as expressed in Equation (2). The calculated results are presented in Figure 5.

(1) $$DS=\frac{\left(t_{2}-t_{1}\right)\times N}{d_{2}-d_{1}}\times C_{1}\times C_{2}$$

where *DS* is dynamic stability of the surface combined body, timing/mm; *d*_1_ indicates the deformation of the surface combined body at *t*_1_ (usually 45 min); *d*_2_ indicates the deformation of the surface combined body at *t*_2_ (usually 60 min); *C*_1_ and *C*_2_ are typically set to 1.0; *N* is the reciprocating rolling speed of the test wheel, typically 42 times/min.

(2) $$ED_{DS}=\frac{DS^{*}-DS}{DS}\times 100$$

where *ER_DS_* is the enhancement ratio of dynamic stability %; DS* is the dynamic stability of the reinforced surface combined body, timing/mm; DS is the dynamic stability of the unreinforced surface combined body, timing/mm.

As illustrated in Figure 5, the dynamic stability of the geogrid-reinforced surface combined body is greater than that of the unreinforced specimen, with corresponding *ER_DS_* values greater than zero. Among all configurations, the AC-13/AC-20 reinforced with CCF presents the maximum *ER_DS_* value of 100.03%. The dynamic stability of the geogrid-reinforced surface layer composite is above 2800 timing/mm, complying with the requirements of the Chinese standard of JTG D50 [59] (Code of China 2017). According to relevant literature [60], the effective contact area ratio is defined as the contact area between the geogrid and asphalt mixture divided by the total interfacial area. Test results indicate that when the effective contact area ratio between the geogrid and asphalt mixture is approximately 0.30, interlayer geogrids still satisfy the interlayer bonding performance requirements of the surface combined body. Meanwhile, geogrids effectively enhance the rutting resistance of the surface combined body. These observations are in good agreement with the findings of Tang et al. [9,60].

Under the same surface combined body conditions, significant variations exist in the contribution of different geogrid types to the *ER_DS_*. Under AC-20/AC-25 conditions, the *ER_DS_* of CCF and GCF reinforced are 88.18% and 66.82%, respectively, indicating that CCF exhibits superior enhancement performance compared to GCF in terms of dynamic stability improvement. In terms of dynamic stability, the CCF value exceeds that of GCF, representing an improvement of 12.80–13.74%. This demonstrates that the geogrid type plays a significant role in enhancing dynamic stability.

Under identical reinforcement conditions, the *ER_DS_* values of AC-20/AC-25 are lower than those of AC-13/AC-20. Under GCF and CCF reinforcement, the *ER_DS_* increases by 13.54% and 13.43%, respectively, when changing from AC-20/AC-25 to AC-13/AC-20, while dynamic stability increases by 40.25% and 41.41%, respectively. Therefore, the appropriate selection of the surface combined body type can enhance the dynamic stability of reinforced surface combined bodies.

### 3.2. Low-Temperature Cracking Resistance

#### 3.2.1. Influence of Geogrid Type and Surface Combined Body Type on Flexural Tensile Strength

**Figure 6:**
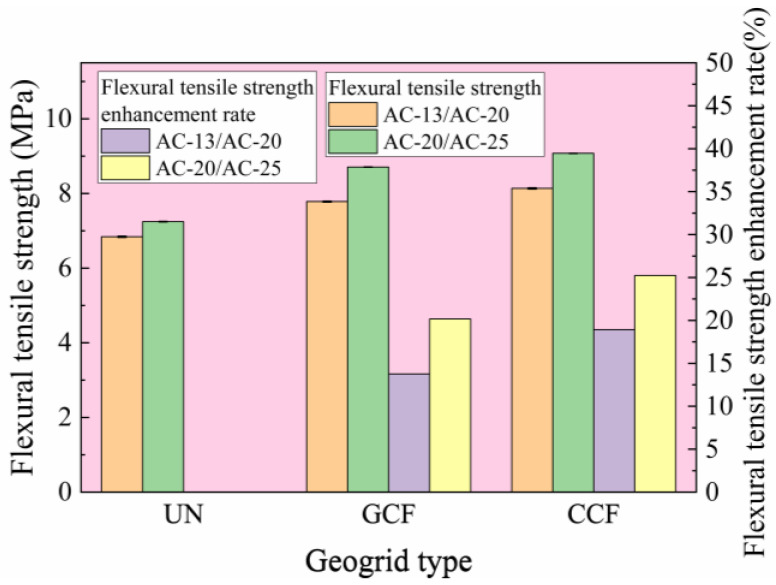
Flexural tensile strength and its enhancement ratio test results.

Figure 6 shows the test results for the flexural tensile strength at failure of different geogrid-reinforced surface combined bodies. To further enhance the comparative analysis of the flexural tensile strength variation and reinforcement effectiveness of two geogrid types (CCF and GCF) within their respective surface combined bodies post-reinforcement, the enhancement ratio of flexural tensile strength (*ERR_B_*) is introduced, as shown in Equation (3). The calculation results are presented in Figure 6.

(3) $${ERR}_{B}=\frac{R_{B}^{*}-R_{B}}{R_{B}}\times 100$$

where $R_{B}^{*}$ is the flexural tensile strength obtained when the reinforced surface combined body fails, MPa; $R_{B}$ is the flexural tensile strength obtained when the unreinforced surface combined body fails, MPa.

It can be seen from Figure 6 that the flexural tensile strength of geogrid-reinforced surface combined bodies consistently exceeds that of unreinforced specimens, with corresponding *ERR_B_* values all exceeding zero. Among these, the AC-20/AC-25 under CCF reinforcement exhibits the highest *ERR_B_*, at 25.24%, indicating that carbon-fiber-based geogrid reinforcement effectively improves flexural tensile strength at failure.

Under the same surface combined body conditions, both the flexural tensile strength and *ERR_B_* of CCF are greater than those of GCF. Under AC-13/AC-20 conditions, when the geogrid transitions from GCF to CCF, the flexural tensile strength and *ERR_B_* increase by 4.53% and 37.47%, respectively. This phenomenon can be explained by the difference in longitudinal rib performance between CCF and GCF. Given that the modulus of carbon fiber is considerably higher than that of glass fiber, CCF exhibits more effective stress diffusion relative to GCF. This reduces the stress at the beam bottom, improves the load-bearing capacity, and enhances the flexural tensile strength at failure.

The flexural tensile strength and *ERR_B_* of AC-13/AC-20 are both lower than those of AC-20/AC-25 under the identical reinforcement conditions. Under CCF reinforcement conditions, when the surface combined body transitions from AC-13/AC-20 to AC-20/AC-25, the flexural tensile strength and *ERR_B_* increase by 11.54% and 33.33%, respectively. The reason may lie in the compatibility between the primary aggregate particle size of the surface combined body and the mesh aperture dimensions [2]. The coarse aggregate for AC-20/AC-25 primarily consists of particles in the 9.5–19 mm range, whereas the coarse aggregate in the AC-13/AC-20 predominantly consists of particles ranging from 4.75 to 13.2 mm. The 9.5–19 mm aggregate particles are similar in size to the mesh openings of the geogrid. The geogrid’s ribs constrain the free movement of aggregate particles, thereby dissipating temperature stresses induced by low-temperature shrinkage.

#### 3.2.2. Influence of Geogrid Type and Surface Combined Body Type on the Maximum Flexural Tensile Strain

**Figure 7:**
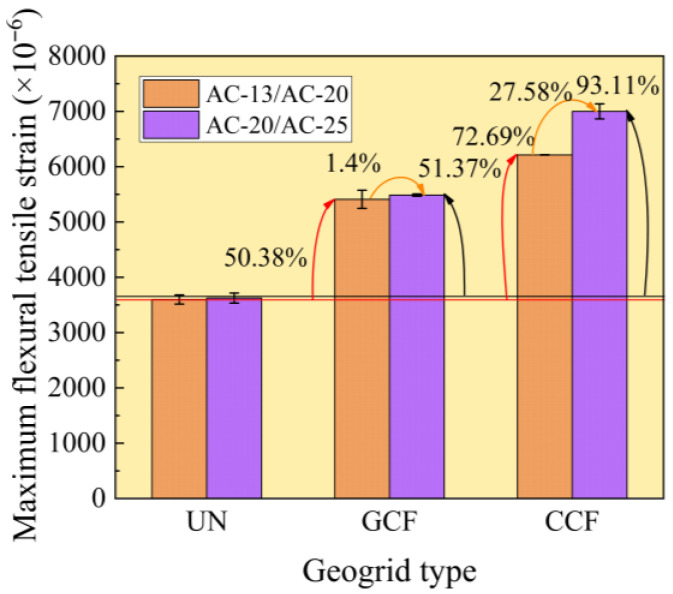
Maximum flexural tensile strain test results.

Figure 7 illustrates the maximum flexural strain test results at failure for different geogrid-reinforced surface combined bodies. As illustrated in Figure 7, all maximum flexural tensile strains are greater than 2800 με, complying with the requirements of the Chinese standard of JTG D50 [59] (Code of China 2017). The maximum flexural tensile strain of the geogrid-reinforced specimen exceeds that of the unreinforced specimen. This indicates that reinforcement with carbon-fiber-based geogrids enhances maximum flexural tensile strain, thereby improving the toughness of the composite structure.

Under identical surface combined bodies, the maximum flexural tensile strain of GCF is consistently lower than that of CCF. Under AC-13/AC-20, when the geogrid transitions from GCF to CCF, the maximum flexural tensile strain is enhanced by 14.84%. This difference is attributed to the variation in longitudinal rib characteristics between CCF and GCF, as longitudinal ribs play a crucial role in both preserving the planar grid structure and enhancing the transverse rib flexural stiffness [57]. The superior longitudinal rib performance of CCF prevents mesh geometric distortion, preserving geogrid modulus, stiffness, and interfacial bond while enhancing reinforcement efficacy.

Under identical reinforcement conditions, the maximum flexural tensile strain of AC-13/AC-20 is consistently lower than that of AC-20/AC-25. Under CCF reinforcement, the maximum flexural tensile strain of the surface combined body increases by 27.58% from AC-13/AC-20 to AC-20/AC-25. The enhancement effect within the surface-layer composite relies on multiple factors, including modulus ratio between layers, gradation, geogrid type, and aggregate type. When particle size closely matches grid dimensions, the interlocking action strengthens, leading to enhanced stress diffusion and improved low-temperature cracking resistance.

### 3.3. Interlayer Shear Performance

#### 3.3.1. Influence of Geogrid Type on Interlayer Shear Strength

**Figure 8:**
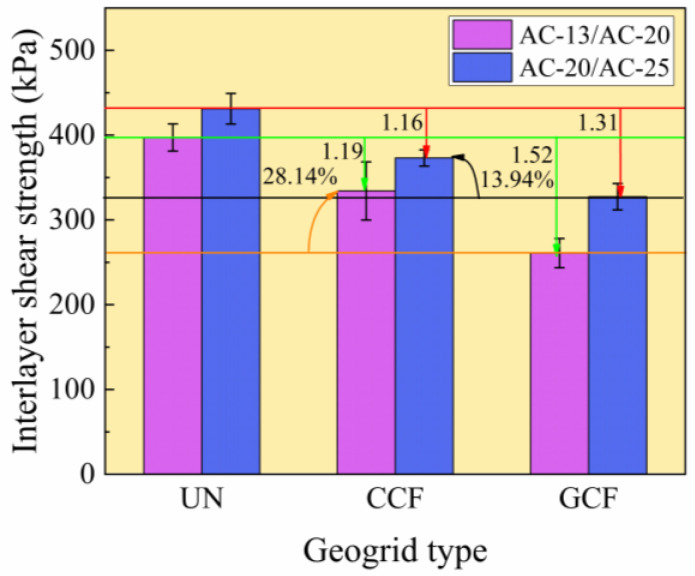
Interlayer shear strength test results.

Figure 8 shows the test results for the interlayer shear strength of various geogrid-reinforced surface combined bodies. As shown in Figure 8, under all surface layer combined bodies, the interlayer shear strength of CCF outperforms that of GCF, exhibiting an improvement ranging from 13.94% to 28.14%. This may be attributed to the brittleness and poor abrasion resistance of glass fiber, leading to inferior performance of the GCF and relatively low interlayer shear strength. The flexibility and high abrasion resistance of carbon fiber enable CCF to effectively embed into the coarse-graded asphalt mixture, reducing wear during shear loading and forming a robust interlayer bond. The above analysis indicates that the interlayer shear strength of CCF and GCF differs only marginally. Consequently, from the perspective of optimizing interlayer shear strength, CCF and GCF may be interchangeable. When performance is paramount, CCF is recommended for achieving optimal interlayer shear strength while enhancing crack resistance. From an economic perspective, GCF is recommended as the interlayer reinforcement material.

Geogrids, when employed as interlayer reinforcement materials, reduce interlayer shear strength. The geogrid type is a contributing factor, with GCF exhibiting a greater reduction than CCF. This is partly attributable to variations in the characteristics of longitudinal and transverse ribs, which reduce the extent of effective contact/bonding between the two layers of asphalt mixture. The interlayer shear strength of unreinforced specimens is 1.16 to 1.19 times that of CCF specimens and 1.31 to 1.52 times that of GCF specimens, confirming the reduction effect. This is attributed to the typically higher peak friction angle between aggregate particles than between aggregate and geogrid [61,62]. Consequently, the shear resistance between aggregates exceeds that between aggregates and the geogrid surface. The unconfined interlayer shear strength values obtained from Leutner shear tests (260.81–373.10 kPa) fell within the range of 100–1700 kPa reported in the literature [27,28,29,63,64], indicating no significant discrepancies. Therefore, geogrids can be expected to provide acceptable interlayer shear performance in practical applications.

#### 3.3.2. Influence of Surface Combined Body Type on Interlayer Shear Strength

Figure 8 also shows that, for all geogrid types, the interlayer shear strength of AC-13/AC-20 is lower than that of AC-20/AC-25. Under unreinforced conditions, the interlayer shear strength of AC-13/AC-20 is approximately 8% lower than that of AC-20/AC-25. Moreover, the interlayer shear strength of AC-13/AC-20 is approximately 11% to 20% lower than that of AC-20/AC-25 under reinforced conditions. This indicates that the surface combined body type exerts a significant influence on interlayer shear strength under geogrid reinforcement conditions [2]. The reason lies in the fact that AC-13 and AC-20 mixtures contain a lower proportion of coarse aggregate compared to AC-20 and AC-25 mixtures. Due to differences in gradation and the physical and mechanical properties of the aggregates, each asphalt mixture produces a distinct texture between layers. AC-20/AC-25 exhibits the most pronounced interfacial roughness, providing favorable conditions for interlayer adhesion. This facilitates mechanical interlocking and bonding between upper and lower layers, forming a robust interlocking structure that enhances interfacial friction. Furthermore, existing research indicates that when the ratio of geogrid aperture size to mean aggregate diameter ranges from 0.96 to 2.0, the interlocking effect at the geogrid-aggregate interface is significantly enhanced, thereby contributing to improved interlayer shear strength [2,62,65,66,67]. The determination process for the specific aggregate size range is outlined below. First, the equivalent particle size of the geogrid is calculated. Second, an initial aggregate size range is established based on existing research. Third, the range is further revised and finalized in accordance with the practical aggregate gradation. For the geogrid aperture size (25 mm) in this study, the specific aggregate size range is 12.5–26 mm. Taking into account the actual gradation, the effective range of specific aggregate sizes is 13.2–26.5 mm. The proportion of aggregates of the specified particle size range within the AC-13/AC-20 and AC-20/AC-25 is 13% and 28%, respectively. When this proportion increases from 13% to 28%, interlayer shear strength improves by 8.5 to 25.6%. Consequently, when selecting carbon-fiber-based geogrids as interlayer reinforcement, compatibility between aggregate gradation and mesh aperture size must be ensured.

## 4. Conclusions

This study investigated the influence of geogrid type and surface combined body type on the high- and low-temperature performance and interlayer shear performance of asphalt pavement structures through a series of rutting tests, low-temperature bending failure tests, and Leutner shear tests, which helps to improve the durability of carbon-fiber-based geogrid reinforced asphalt pavement.

(1) Carbon-fiber-based geogrid reinforcement enhances the rutting resistance of the asphalt surface combined body, with CCF reinforcement demonstrating superior rutting resistance compared to GCF reinforcement. In this study, the maximum rutting depth of GCF reinforcement is 1.08 times that of CCF reinforcement. Compared with GCF reinforcement, the dynamic stability of CCF reinforcement increases by 12.80–13.74%.

(2) Carbon-fiber-based geogrid reinforcement improves the low-temperature cracking resistance of the asphalt surface combined body, with CCF reinforcement demonstrating superior low-temperature cracking resistance compared to GCF reinforcement. In this study, under the AC-13/AC-20 conditions, the flexural tensile strength and *ERR_B_* of CCF reinforcement increase by 4.53% and 37.47%, respectively, compared to GCF reinforcement.

(3) Carbon-fiber-based geogrid reinforcement reduces the interlayer shear performance of the asphalt surface combined body. However, CCF reinforcement exhibits superior interlayer shear performance compared to GCF reinforcement, demonstrating an improvement of 13.94–28.14%. When optimizing interlayer shear strength, CCF and GCF can replace each other, but must meet other performance requirements.

(4) Surface combined body type is a factor that affects the high- and low-temperature performance and interlayer shear performance of reinforced surface combined body. In this study, the maximum flexural tensile strain and dynamic stability of AC-20/AC-25 are higher than those of AC-13/AC-20, with an increase of 27.58% and 40.25%, respectively. The interlayer shear strength of AC-13/AC-20 is approximately 11% to 20% lower than that of AC-20/AC-25 under reinforced conditions.

For the reinforced surface combined body, this experiment is only an indoor study, focusing on its high- and low-temperature performance and interlayer shear performance, which has certain limitations. The next step will be to conduct research in the following areas. Such as the high- and low-temperature performance of reinforced surface combined bodies, in situ, alongside their interlayer shear behavior. The influence of carbon fiber geogrid size and specific particle size (such as 13.2–26.5 mm) percentage in aggregates on the high- and low-temperature performance and interlayer shear performance of surface combined bodies, and the fatigue resistance of reinforced surface combined bodies.

## Figures and Tables

**Table 1:** Technical specifications of geogrids.

Geogrid Properties		GCF	CCF
Type		Carbon Fiber-Fiber Glass	Carbon Fiber
Ultimate elongation(%)	Transverse	≤2	≤2
	Longitudinal	≤3	≤2
Ultimate tensile strength (kN/m)	Transverse	80	80
	Longitudinal	50	80
Tensile stiffness (kN/m)		3380	3620
Thickness (mm)		0.6	0.6
Aperture size (mm × mm)		25 × 25	25 × 25

**Table 2:** Technical specifications and material properties of SBS-modified asphalt.

Type	Measured Value	Standardized Requirement
Softening point (°C)	64.4	≥60
Penetration, 25 °C, 5 s, 100 g (0.1 mm)	57.8	30~60
Brinell viscosity 135 °C (Pas)	1.9	≤3
Ductility, 5 cm/min, 10 °C (cm)	25.1	≥20
Flash point (°C)	270	≥230

**Table 3:** Technical specifications of PCR cationic emulsified asphalt.

Type	Cationic Rapid Setting Emulsified Asphalt	
Identification of cationic property		Positive
Content of residual binder (%)		50.1
Viscosity (Pas)	Engra viscosity E_25_	10
	Standard viscosity of asphalt C_25, 3_	25
Sieve test (%)		0.09
Storage stability (%)	1 d	<0.5
	5 d	<2

**Table 4:** Asphalt-aggregate ratio of asphalt mixture.

Asphalt Mixture	AC-25	AC-20	AC-13
Asphalt-aggregate Ratio (%)	4.8	4.3	3.7

## Data Availability

The data used to support the findings of this study are available from the corresponding author upon request.

## Abbreviations

ANOVA	Analysis of variance
CCF	Carbon fiber geogrid
GCF	Glass/carbon fiber composite geogrid
AC	Dense-graded asphalt concrete mixture
SBS	Styrene-butadiene-styrene block copolymer
*ER_DS_*	Enhancement ratio of dynamic stability
*ERR_B_*	Enhancement ratio of flexural tensile strength
